# Blood supply to the cranial cavity in the patagonian mara (*Dolichotis patagonum*)

**DOI:** 10.1007/s11259-023-10113-1

**Published:** 2023-03-28

**Authors:** Maciej Zdun, Oleg P. Melnyk, Oleksii O. Melnyk, Maria Nabzdyk

**Affiliations:** 1 Department of Animal Anatomy, Poznan University of Life Sciences, Wojska Polskiego 71C, Poznań, 60-625 Poland; 2 Department of Basic and Preclinical Sciences, Nicolaus Copernicus University, Lwowska 1, Toruń, 87-100 Poland; 3 Department of Animal Anatomy, Histology and Pathomorphology, National University of Life and Environmental Sciences of Ukraine, Heroiv Oborony Str.15, Kyiv, 03041 Ukraine

**Keywords:** Angiology, Brain arteries, Brain vascularization, Patagonian Cavy

## Abstract

Rodents are the most numerous order of mammals. The literature presents information on the arterial circle of the brain in capybara, the guinea pig of the family *Caviidae* and many other not so closely related rodent species. Information on the blood supply to the brain is often incomplete and focuses on one pathway in a broader comparative aspect. The supply of oxygen and nutrients to the brain is very important for its proper functioning. The aim of this study is to describe the pathways supplying blood to the cranial cavity and to describe the arterial circle of the brain in the Patagonian mara. The study was conducted on 46 specimens using two methods. The first of them used a stained solution of the chemo-setting acrylic material. The second one, the colored liquid LBS 3060 latex. The arterial circle of the brain is a heart-shaped structure. It is formed by rostral cerebral arteries, caudal communicating arteries and the basilar artery. Blood supplies the arterial circle of the brain in three ways. First one is the basilar artery, which originates from the vertebral arteries. The second one is the internal carotid artery which joins a branch from the external ophthalmic artery. The third is the internal ophthalmic artery, which branches from the external ophthalmic artery.

## Introduction

Rodents are the most numerous order of mammals. The Patagonian mara (*Dolichotis patagonum*) belongs to the suborder *Hystricomorpha*, family *Caviidae*, and subfamily *Dolichotinae*. The Patagonian Mara is the second largest representative of the *Caviidae* family. It is an endemic species of the open grasslands and steppes of Argentina. It is commonly found between 28°S and 50°S. (Chillo et al. 2010).

The literature presents information on the arterial circle of the brain in the capybara, guinea pig, and Spix’s yellow-toothed cavy of the family *Caviidae* (Costa et al. 2017; Ocal and Ozer 1992; Reckziegel et al. 2001) and many other not so closely related rodent species. Information on the blood supply to the brain is often incomplete and focuses on one pathway in a broader comparative aspect.

The supply of oxygen and nutrients to the brain is very important for its proper functioning. Fainting or even death could result from a congested vessel (Tzeng and Ainslie 2014). Rodents are a group of animals that are often used in animal experiments. Considering that fact, information from this paper may be helpful not only in veterinary medicine but also for scientists using animals for research.

The aim of this study is to describe the pathways supplying blood to the cranial cavity and to describe the arterial circle of the brain in the Patagonian mara.

## Materials and methods

### Animals

The study was conducted on 46 adults (22 male and 24 female) Patagonian mara of weight ranging from five to nine kilograms and aged three to twelve years. The analyzed specimens were delivered as post-mortem material from zoological gardens and private breeders. No animals were sacrificed for this study.

### Methods

The specimens were prepared using two methods. The thirty-eight specimens were studied by injecting the bilateral common carotid arteries with a stained solution of the chemo-setting acrylic material Duracryl® Plus (SpofaDental) by hand pressure. When the material was polymerized, the specimens were macerated enzymatically (Persil—Henkel) at a temperature of 40^o^C for 30 days. The process resulted in casts of arteries of the head and encephalic base on a skeletal scaffold. The second method, used for eight specimens, colored liquid LBS 3060 (Synthos) latex was introduced into the bilateral common carotid arteries by hand pressure. After curing in 5% formalin solution, the blood vessels were prepared manually using surgical instruments. This way, blood vessels on the animal’s soft tissues were obtained. Both methods are complementary, allowing the tracing of arteries in relation to bone and soft tissue. The skulls were opened using an oscillating saw. The vessels which provided blood to the brain were measured close to the point of entry into the arterial circle of the brain using calipers. From all measurements, the average value presented in the text was calculated.

### Results

**Fig. 1:**
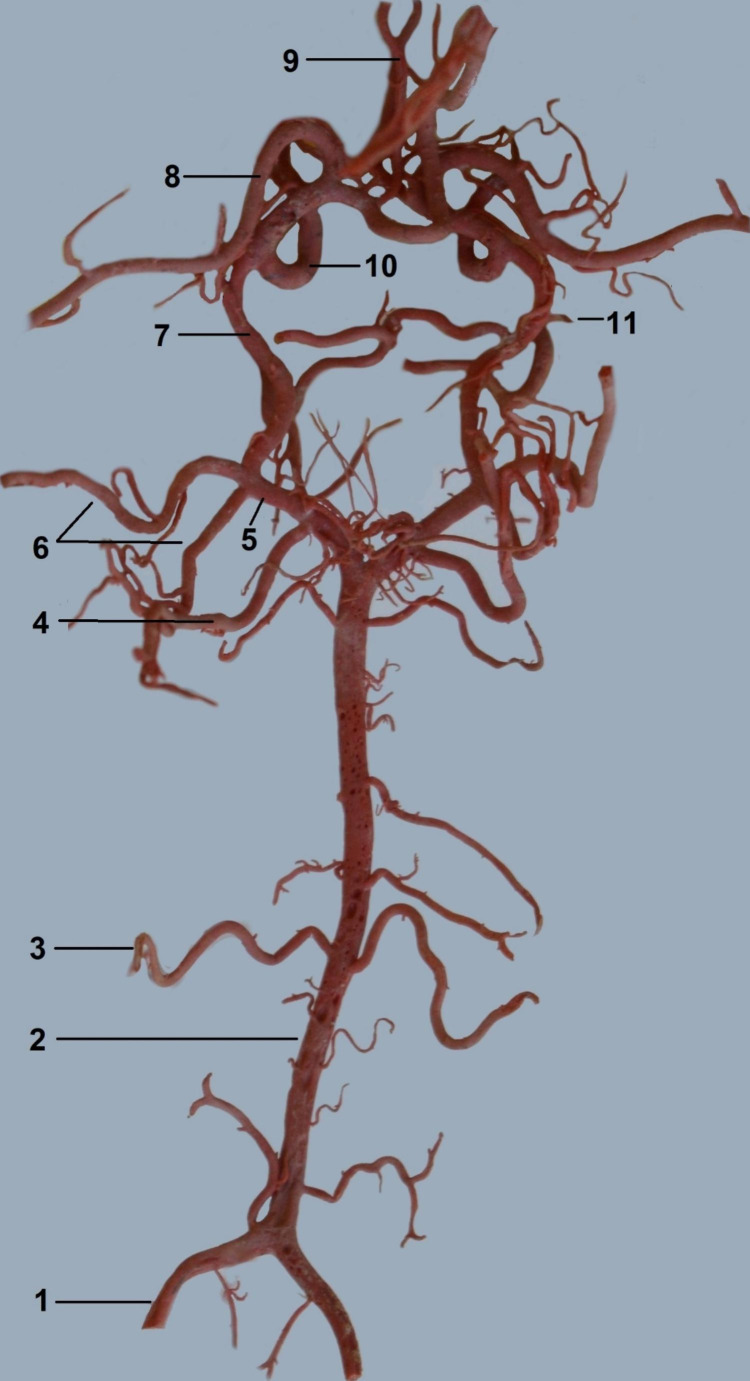
The arterial circle of the brain. Corrosion cast 1—the vertebral artery 2—the basilar artery 3—the caudal cerebellar artery 4—the rostral cerebellar artery 5—the caudal communicating artery 6—the caudal cerebral arteries 7—the rostral cerebral artery 8—the middle cerebral artery 9—the internal ethmoidal artery 10—the internal ophthalmic artery 11—the rostral choroid artery

The arterial circle of the brain (Fig. 1) is a heart-shaped structure.

**Fig. 2:**
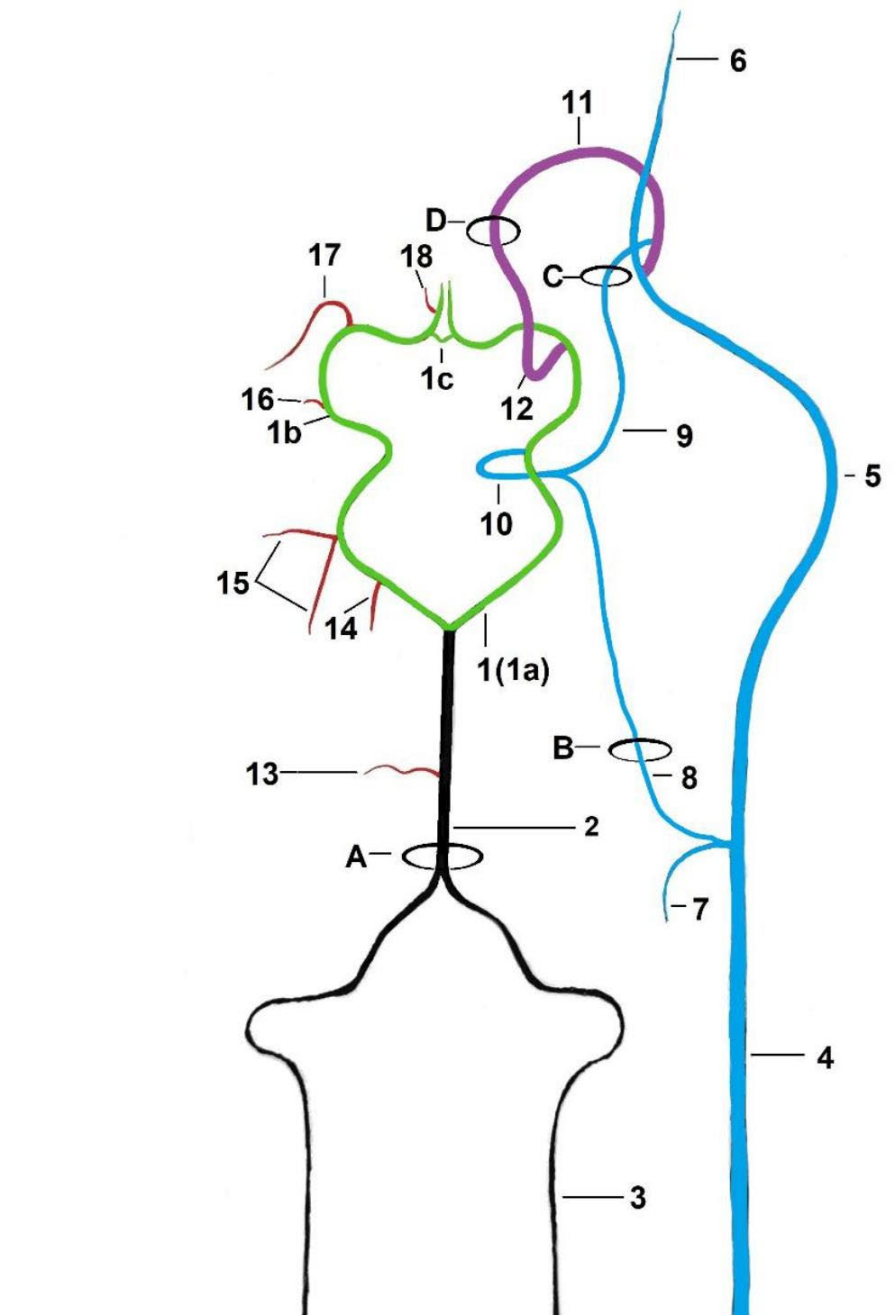
The arterial circle of the brain with blood supply pathways. The right side of the figure shows the pathways by which blood enters the brain. The left side of the figure shows the branches from the arterial circle of the brain. Green—the arterial circle of the brain; black—the first source of blood (the basilar artery); blue—the second source of blood (the common trunk of the internal carotid artery and the branch from the external ophthalmic artery); purple—the third source of blood (the internal ophthalmic artery); red—vessels branching off from the arterial circle of the brain. The width of the dash marks reflects the width of the vessel lumen 1—the arterial circle of the brain, 1a—the caudal communicating artery, 1b—the rostral cerebral artery, 1c—the rostral communicating artery, 2—the basilar artery, 3—the vertebral artery, 4—the common carotid artery, 5—the external carotid artery, 6—the maxillary artery, 7—the occipital artery, 8—the internal carotid artery, 9—branch from the external ophthalmic artery, 10—common trunk of the internal carotid artery and the branch from the external ophthalmic artery, 11—the external ophthalmic artery, 12—the internal ophthalmic artery, 13—the caudal cerebellar artery, 14—the rostral cerebellar artery, 15—the caudal cerebral arteries, 16—the rostral choroid artery, 17—the middle cerebral artery, 18—the internal ethmoid artery, A—the foramen magnum, B—the carotid artery canal, C—the orbitorotundum foramen, D—the optic canal

It is formed by rostral cerebral arteries, caudal communicating arteries, and the basilar artery. The basilar artery joins the caudal communicating arteries caudally. Blood supplies the arterial circle of the brain in three ways (Fig. 2).

From the caudal side, blood flows via the basilar artery. This vessel is formed by the fusion of the bilateral vertebral arteries. In the cervical section of the spine, the vertebral artery on both sides of the body follows the atlas cranially, passing through the foramen of the transverse process of the subsequent vertebrae. Then, it enters the atlas through the lateral vertebral foramen and joins the identical vessel on the other side. This gives rise to the odd vertebral artery, which lies in the body’s midline. Throughout its course, the basilar artery has the same diameter (1,05 mm ± 0,25). Near the middle of its length, the caudal cerebellar artery (0,2 mm ± 0,1) branches off from the basilar artery. It is a single vessel on each side of the body that supplies the cerebellum’s caudal part.

In addition, small arteries to the medulla oblongata branch off from the basilar artery. It then joins the caudal communicating arteries (0,75 mm ± 0,15). The diameter of these vessels is significantly smaller than that of the basilar artery. Each caudal communicating artery branched off the rostral cerebellar artery (0,3 mm ± 0,1). It is a larger diameter vessel than the caudal cerebellar artery. The rostral cerebellar artery supplies a larger cerebellum area on the rostral side. Further vessels branching off from the caudal communicating artery are the caudal cerebral arteries, supplies the caudolateral part of the cerebrum. These are double vessels on each side of the body, one of which has a larger diameter than the other (0,45 mm ± 0,1; 0,3 mm ± 0,15). They usually branch off together. In seven individuals unilaterally and three individuals bilaterally, these vessels branch off separately although very close to each other.

**Fig. 3:**
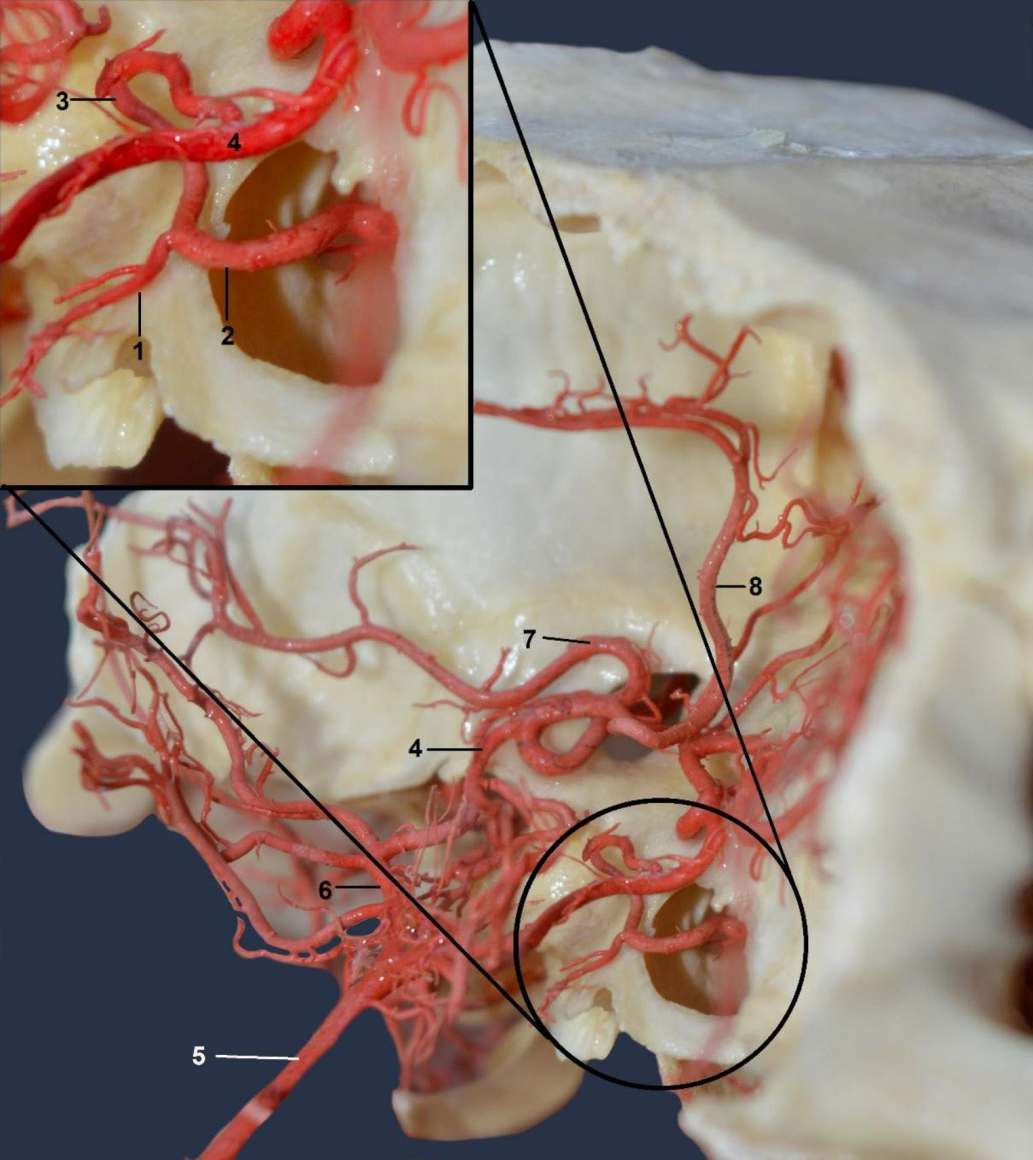
The arterial circle of the brain on the bone scaffold. Dorsolateral view. In closer: point of connection of the internal carotid artery with the branch from the external ophthalmic artery. Corrosion cast 1—the internal carotid artery (caudal part broken off during bone removal to take the photo) 2—branch from the external ophthalmic artery passing through the orbitorotundum foramen 3—final section of the internal carotid artery 4—the left and right rostral cerebral artery 5—the basilar artery 6—the caudal communicating artery 7—the middle cerebral artery 8—the rostral cerebral artery, vertical part in the longitudinal fissure of the brain

Halfway along the length of the arterial circle of the brain, the internal carotid artery (0,15 mm ± 0,05) joins it. It branches off from the common carotid artery by a common trunk with the occipital artery. The internal carotid artery enters the cranial cavity through the carotid artery canal. Before joining the arterial circle of the brain, it joins a branch from the external ophthalmic artery (0,2 mm ± 0,15) (Fig. 3).

This branch enters the *foramen orbitorotundum* into the cranial cavity. After connecting with a branch of the external ophthalmic artery, the internal carotid artery forms an arc, resembling a U on its side, and then joins the arterial circle of the brain. Considering the small diameter of this vessel, the significance of this blood way seems marginal.

From the rostral side, blood flows into the arterial circle of the brain via the internal ophthalmic artery (1,05 mm ± 0,3), which branches from the external ophthalmic artery. The external ophthalmic artery, branches off from the maxillary artery. After giving off branches to the eyeball and the external ethmoidal artery, passes through the optic canal and joins the rostral cerebral artery as the internal ophthalmic artery. The middle cerebral artery branches off from the rostral cerebral artery. It is a strong vessel (0,6 mm ± 0,15) supplying the anterolateral part of the brain. Then, more rostrally, a thin rostral communicating artery joins the bilateral rostral cerebral arteries in 38 specimens. The presence of this vessel results in a closed arterial circle of the brain on the rostral side. Moreover, a thin internal ethmoidal artery branches off from the rostral cerebral artery. The internal ethmoidal artery leads to the cribriform plate. Finally, the rostral cerebral artery (0,45 mm ± 0,15) changes direction and lays in the longitudinal fissure of the brain, revascularizing the medial surface of the brain.

## Discussion

For many rodents, the only source of blood for the brain is the very strong basilar artery, which originates from the vertebral arteries. Such a model of cerebral vascularisation has been described in the ground squirrel (Aydin et al. 2009), porcupine (Aydin et al. 2005), chinchilla (Kuchinka 2017), pacarana (Freitas et al. 2018), capybara (Reckziegel et al. 2001), red squirrel (Aydin 2008) and nutria (Azambuja et al. 2018). The process of atrophy of the internal carotid artery in the capybara has been carefully analyzed. At six months of age, the animal is an unobstructed vessel supplying blood to the brain. It no longer performs this function in animals at 12 months of age. The lumen is collapsed, and the layer of vascular smooth muscle cells is almost entirely replaced by connective tissue. In these older animals, a twofold increase in the diameter of the basilar artery was observed (Steele et al. 2006). In some species, a very thin internal carotid artery connects to the arterial circle of the brain. This vessel does not serve as a viable source of blood supply to this vascular area due to the very small lumen of the vessel. The presence of the internal carotid artery in this form has been described in the degu (Brudnicki et al. 2014) and guinea pig (Ocal and Ozer 1992). In a few species, which include agouti, Egyptian spiny mouse, mouse, Mongolian gerbil, rat, hamster and vole, there is a well-developed internal carotid artery that, together with the basilar artery, contributes to the supply of blood to the brain (Brown 1966; Bugge 1970; Ghanavati et al. 2014; Kuchinka et al. 2008; Silva et al. 2016; Szczurkowski et al. 2007). In the Mongolian gerbil, Egyptian spiny mouse and mouse the caudal communicating artery is not present. Consequently, the basilar artery supplies the midbrain and hindbrain, whereas the internal carotid artery supplies the forebrain and diencephalon (Firbas et al. 1973; Ghanavati et al. 2014; Kuchinka et al. 2008; Szczurkowski et al. 2007). In the Patagonian mara, we observe a well-developed basilar artery, which is also the case in all the rodents cited above. In addition, the internal carotid artery also joins the arterial circle of the brain. A feature that distinguishes the Patagonian mara from the degu and guinea pig is that before this artery joins the arterial circle of the brain, it is joined by a branch from the external ophthalmic artery. The internal ophthalmic artery joins the rostral cerebral artery. Given the diameter of the vessels, this is an important source of blood. A large-diameter internal ophthalmic artery has been described in the guinea pig (Kuchinka 2018).

The shape of the arterial circle of the brain in the analysed species differs from the shapes of this structure in other rodents. It is most similar to the porcupine (Aydin et al. 2005). In most rodent species, it resembles an ellipse, where the lateral parts are elongated and the rostral and caudal parts assume an arched shape (Aydin 2008; Aydin et al. 2009; Azambuja et al. 2018; Brudnicki et al. 2014; Freitas et al. 2018; Ghanavati et al. 2014; Kuchinka 2017; Ocal and Ozer 1992; Reckziegel et al. 2001; Silva et al. 2016; Szczurkowski et al. 2007).

The point of branching off of an individual vessel from the arterial circle of the brain can be species-variable. Only in the ground squirrel the caudal cerebellar artery branch off from the vertebral artery described (Aydin et al. 2009). In other species, this artery diverged from the basilar artery. The middle cerebellar artery branching off from the basilar artery was observed in the capybara, ground squirrel, porcupine and pacarana (Aydin et al. 2005, 2009; Freitas et al. 2018; Reckziegel et al. 2001). In the Patagonian mara, the presence of this vessel was not observed. The rostral cerebellar artery branching off from the basilar artery was observed in the ground squirrel (Aydin et al. 2009), agouti (Silva et al. 2016), red squirrel (Aydin 2008), Mongolian gerbil (Kuchinka et al. 2008), capybara (Reckziegel et al. 2001), rat (Brown 1966), Egyptian spiny mouse (Szczurkowski et al. 2007) and mouse (Ghanavati et al. 2014). In contrast, in the porcupine (Aydin et al. 2005), degu (Brudnicki et al. 2014), pacarana (Freitas et al. 2018), guinea pig (Ocal and Ozer 1992), chinchilla (Kuchinka 2017), and the nutria (Azambuja et al. 2018) this vessel branched off from the caudal communicating artery. From the rostral side, the arterial circle of the brain can be an open or closed structure. The presence of the rostral communicating arteries results in a closed arterial circle of the brain. This type of structure is in the capybara (Reckziegel et al. 2001), porcupine (Aydin et al. 2005), degu (Brudnicki et al. 2014), pacarana (Freitas et al. 2018), Mongolian gerbil (Kuchinka et al. 2008), in addition occasionally in the rat (Brown 1966) and red squirrel (Aydin 2008). The absence of this vessel was found in the ground squirrel (Aydin et al. 2009), in 60% of nutria (Azambuja et al. 2018), and 77% of chinchillas (Kuchinka 2017). The point of branching off the remaining vessels is analogous in all species.

## Conclusions

In the Patagonian mara the well-developed basilar artery was observed. In addition, the analysis showed an extremely thin internal carotid artery. Moreover, the well-developed internal ophthalmic artery and the connection between the internal carotid artery and the external ophthalmic artery was observed. In the shape of the arterial circle, similarities can only be found in the porcupine, which is, however, a representative of a different family.

## Data Availability

The data used to support the findings of this study are available from the corresponding author upon reasonable request.
